# External Airborne-agent Exposure Increase Risk of Digestive Tract Cancer

**DOI:** 10.1038/s41598-020-65312-6

**Published:** 2020-05-25

**Authors:** Wanhyung Lee, Jihyun Kim, Sung-Shil Lim, Yangwook Kim, Yeon-Soon Ahn, Jin-Ha Yoon

**Affiliations:** 1 Department of Occupational and Environmental Medicine, Gil Medical Center Gachon University College of Medicine, Incheon, Republic of Korea; 2 The Institute for Occupational Health, Yonsei University College of Medicine, Seoul, Republic of Korea; 3 Graduate School of Public Health, Yonsei University College of Medicine, Seoul, Republic of Korea; 4 Department of Preventive Medicine, Wonju College of Medicine, Yonsei University, Wonju, Korea; 5 Department of Preventive Medicine, Yonsei University College of Medicine, Seoul, Republic of Korea

**Keywords:** Cancer epidemiology, Risk factors

## Abstract

Previous studies have suggested that in addition to respiratory system cancers, exposure to external airborne agents (EAAs) may also affect the risk of digestive tract cancer. However, previous epidemiological studies have been limited. To clarify this relationship, we conducted a Workers’ Korea National Health Insurance Service cohort study. The EAA exposure group comprised participants who had ever visited a hospital as an inpatient for ‘lung diseases due to external agents’. The reference population comprised men from the general working population. The EAA exposure group and reference group included a total of 98,666 and 79,959,286 person-years, respectively. Age-adjusted standardized incident rates (SIRs) with 95% confidence intervals (95%CI) were calculated for each 5-year age stratum. The SIR (95% CI) of EAA exposure was 1.30 (1.19–1.38) for all digestive tract cancers. The highest risk associated with EAA exposure was observed for oral cancer, followed by esophageal and stomach cancers [SIRs (95%CI): 3.96 (3.02–4.78), 3.47(2.60–4.25), and 1.34(1.17–1.47), respectively.] These statistically significant associations did not be attenuated in a subgroup analysis using logistic regression adjusted for age, smoking and alcohol consumption. Our findings suggest that EAA exposure should address risk reduction of both digestive tract and respiratory system cancers.

## Introduction

Previous studies of the human health effects of external airborne agent (EAA) which were included aerosol, gas, vapor, mist, fume, dust, or smoke exposure have focused on the respiratory system^1–3^, given the assumption that such exposure primarily causes respiratory system disease. Although EAA exposure mainly occurs via the respiratory system, humans face other possible sites of exposure, namely the digestive system. The digestive system may be exposed to EAAs via several inhalation and ingestion mechanisms. First, agents filtered in the nose or exhaled from the upper respiratory tract may be swallowed^4^. Second,, reduced sphincter tone around the esophageal orifice could directly allow the accidental swallowing of EAAs^5^. Third, food, skin, or clothing may be contaminated by EAAs^4^. Finally, an intuitive approach reveals that the origins of the gastrointestinal tract and respiratory system occur in shared structures, such as the oral cavity and pharynx. In summary, these mechanisms identify the gastrointestinal tract as a potential site of EAA exposure, with the potential for inflammatory, immunologic, or even oncogenic responses similar to those observed in the respiratory system^6^.

To date, little is known about the relationship between EAA exposure and the risk of digestive tract cancers. Notably, a previous well-designed cohort study found that esophageal and stomach cancer were more closely correlated with EAA exposure when compared with lung cancer, although the authors did not control for alcohol consumption^7^. Nevertheless, these results encouraged subsequent epidemiological studies. Although later research also demonstrated the effects of EAAs on gastrointestinal tract cancers^8,9^, little epidemiologic evidence is available regarding the risk of cancer throughout the digestive system and potential confounding factors.

A recent study found a high risk of all digestive tract cancers, and particularly of esophageal, stomach, and liver cancer, among participants exposed to external airborne carcinogens after controlling for a smoking habit^10^. However, the authors did not control for alcohol consumption and thus could not fully discuss the potential biological effects of EAA exposure on the increased risk of liver cancer. In a case-control study of the link between EAA exposure and the risk of digestive cancer^11^, the authors found no significant effects after controlling for alcohol consumption and smoking habits; however, that study may have been limited by a relatively small sample size.

In light of the limits of the above-described studies with respect to limited organ-specific outcome data, a lack of information about confounding factors, and a small sample size, this study aimed to determine the whole-digestive system cancer risk by organ (oral cavity to anus), using national follow-up data from the entire population of the Republic of Korea. Our subgroup analysis, which controlled for smoking and alcohol consumption, and the inclusion of a wash-out period in our study design have provided scientific evidence supporting a link between EAA and the risk of digestive tract cancer.

## Results

A male population with a total of 79,959,286 person-years was included in this study. The EAA exposure group included a total of 98,666 person-years (Supplementary Table 1). According to the Korea national health insurance service (NHIS) database, the most common cause of EAA exposure during the study period (2006–2015) was J69, Pneumonitis due to solids and liquids (27,463 person-years, 27.8%) followed by J68, Respiratory conditions due to inhalation of chemicals, gases, fumes, and vapors (22,795 person-years, 23.1%); J64, Unspecified pneumoconiosis (12,428 person-years, 12.6%); and J60, Coalworker pneumoconiosis (11,812 person-years, 12.0%) (Table 1).

**Table 1:** Hospital facility visits information of respiratory disease from external airborne. Agent exposed group according to ICD-10 from 2006 to 2015.

	Person-year	%
J60-J70 Lung diseases due to external agents	98,666	100.0
J60 Coalworker pneumoconiosis	11,812	12.0
J61 Pneumoconiosis due to asbestos and other mineral fibres	3,008	3.1
J62 Pneumoconiosis due to dust containing silica	3,122	3.2
J63 Pneumoconiosis due to other inorganic dusts	1,114	1.1
J64 Unspecified pneumoconiosis	12,428	12.6
J65 Pneumoconiosis associated with tuberculosis	963	1.0
J66 Airway disease due to specific organic dust	597	0.6
J67 Hypersensitivity pneumonitis due to organic dust	10,487	10.6
J68 Respiratory conditions due to inhalation of chemicals, gases, fumes and vapours	22,795	23.1
J69 Pneumonitis due to solids and liquids	27,463	27.8
J70 Respiratory conditions due to other external agents	4,877	4.9

**Figure 1:**
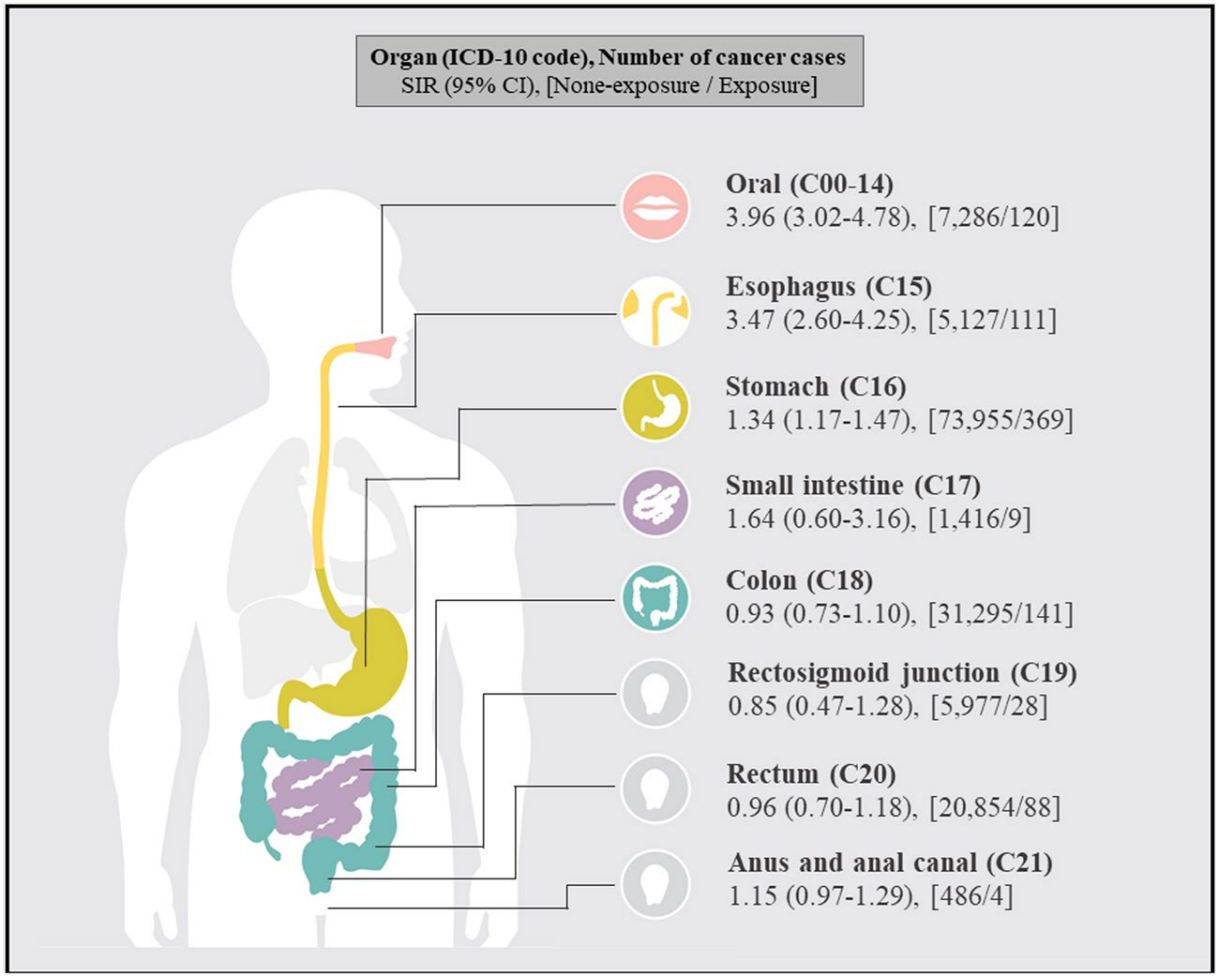
Age-standardized incidence ratio (SIR) and 95% confidence intervals (95% CIs) of malignant neoplasm of digestive systems on external airborne agent exposure group.

We observed a statistically significantly increased risk of digestive cancer among the EAA exposure group, as shown in Table 2 and Fig. 1, with an age-standardized incidence ratio (SIR) (95% confidence interval (CI)) of 1.30 (1.19–1.38) for the risk of all digestive cancers. A stratified analysis yielded SIR (95% CI) values of 1.22 (1.12–1.30) for all gastrointestinal and hepatobiliary tract cancers (C15–26) and of 1.28 (1.15–1.37) for all gastrointestinal tract cancers (C15–21). Among digestive organs, the highest risk was observed for oral cancer, followed by esophageal and stomach cancer, with respective SIRs (95% CI) of 3.96 (3.02–4.78), 3.47 (2.60–4.25), and 1.34 (1.17–1.47). EAA exposure was not associated with significant risks of cancer in the remaining organs, including those in the hepatobiliary tract.

**Table 2:** Age-standardized incidence ratio (SIR) and 95% confidence intervals (CI) of cancer of digestive systems among external airborne agent exposure group.

Cancer type (ICD-10)	Cases	SIR	95% CI
All (C00-26)	**192,695**	**1.30**	**(1.19–1.38)**
Oral (C00-14)	**7,406**	**3.96**	**(3.02–4.78)**
Gastrointestinal and hepatobiliary tract (C15-26)	**185,854**	**1.22**	**(1.12–1.30)**
Gastrointestinal tract (C15-21)	**130,035**	**1.28**	**(1.15–1.37)**
Hepatobiliary tract (C22-26)	58,295	1.15	(0.97**–**1.29)

Tables 3 and 4 present the results from subgroup analyses of medical screening data from study participants. Although the drinking statuses were similar when the workers were stratified by EEA exposure, a significantly higher proportion of current smokers was observed among patients without EEA exposure, compared to those with EEA exposure (p < 0.0001), as shown in Table 3. In a logistic regression analysis, the risks of oral, esophageal, and stomach cancer were significantly increased even after controlling for age, smoking, and drinking status, as shown in Table 4 [Odds Ratio (OR) (95% CI) = 1.96 (1.28–3.00) for oral cancer, 2.45 (1.15–5.20) for esophageal cancer, and 1.45 (1.07-1.96) for stomach cancer.]

**Table 3:** Smoking and drinking status according to external airborne agent exposure group status from medical examination information among study participants (n = 300,290).

	Male workers with external airborne agent exposure group		P-value
	No, n (%)	Yes, n (%)	
Smoking			
None and past smokers	193,325 (64.6)	632 (71.4)	<.0001
Current smokers	106,080 (35.4)	253 (28.6)	
Alcohol consumption			0.5857
None and mild drinking	255,856 (85.4)	762 (86.1)	
Heavy drinking	43,549 (14.6)	123 (13.9)

**Table 4:** Results of logistic regression for cancer of digestive systems among external airborne agent exposure group using data from medical examination information among study participants (n = 300,290).

Cancer type (ICD-10)	OR	95% CI
All (C00-26)	**1.36**	**(1.13-1.66)**
Oral (C00-14)	**1.96**	**(1.28-3.00)**
Gastrointestinal and hepatobiliary tract (C15-26)	**1.28**	**(1.04-1.57)**
Gastrointestinal tract (C15-21)	**1.38**	**(1.10-1.74)**
Esophagus (C15)	**2.45**	**(1.15-5.20)**
Stomach (C16)	**1.45**	**(1.07-1.96)**
Small intestine (C17)	1.45	(0.20-10.38)
Colon (C18)	1.20	(0.79-1.82)
Rectosigmoid junction (C19)	1.24	(0.51-3.00)
Rectum (C20)	1.67	(0.95-2.64)
Anus and anal canal (C21)	3.12	(0.98-9.81)
Hepatobiliary tract (C22-26)	0.94	(0.62-1.41)

## Discussion

The findings of our study demonstrate that workers with lung diseases consequent to EAA exposure face a significantly increased risk of digestive system cancer. Surprisingly, the risk of digestive tract cancer risk exhibited an increasing trend according to the assumed level of EAA exposure (from the upper to lower digestive tract), such that significant increases were observed in the risks of oral, esophageal, and stomach cancers. However, similar relationships were not observed for cancers in other digestive organs ranging from the small intestine to the anus and anal canal and including the hepatobiliary tract. This trend suggests that exposure to EAAs may occur via both inhalation and ingestion. Furthermore, we did not observe significant attenuation of this increased risk of cancer following EAA exposure even after controlling for smoking and alcohol consumption, which are considered strong risk factors for oral and esophageal cancers. These results suggest that EAA exposure via ingestion is more strongly carcinogenic than previously expected. Accordingly, professionals who aim to prevent or treat cancers should address the risk of simultaneous EAA exposure via the respiratory and digestive systems.

Result from current analysis are in line with those of previous studies^8,9,12^. There were dose-response relationship between environmental airborne dust exposure level and risk of risk of death attributed to gastric cancer. Among workers, higher occupational dust exposure industry group showed higher risk of gastric cancer. Our data showed increased risk of oral and gastrointestinal tract cancer, trend was found from oral to only gastric cancer. It’s provided the extended scope of effect from EAA to human digestive system. Previous study indicated occupational dust particles are considering that cause inflammatory reactions related with cancer in both respiratory and digestive organ especially in upper tract^13^. Furthermore, similarly to our results, only high occupational dust exposed group showed statistically significant relationship with cancer in not whole digestive system, but stomach^14^.

However, this result has not previously been described. There was partially significant association between occupational dust exposure and GI tract cancer among current and heavy smokers after stratified by smoking status^15^. According to previous research for stomach cancer risk according to industrial type showed increased risk of stomach cancer among workers in both dust and heat exposed industry (cooks or food and related products machine operator), workers only dust exposed industry were not even considered as severe dust exposed industry (miners and quarry)^16^. There were might be combined effect of EEA with other co-exposed occupational hazardous factors or health behavioral. It indicated that it is important to understand various exposure route and interactive effects of EEA on human health.

According to the Organization for Economic Cooperation and Development, more than 33% of cancer deaths among men in 2013 were caused by cancers of the digestive organs^17^. Generally, a large proportion of the population is exposed to several EAAs via inhalation and ingestion while they leave or work, and both inhaled and ingested EAAs are widely considered to be health hazards^18^. However, little is known about the latter route of exposure and its relationship with digestive cancer. The various types of EAA can be categorized by composition or origin^19,20^ and yield diverse clinical outcomes^21,22^. Regardless, exposure to an EAA can induce acute or chronic inflammation characterized by inflammatory cytokines^18^ or trigger a mutation in an oncogene^23,24^. These processes play a key role in irreversible fibrotic changes or oncogenic mutations in human organs exposed to EAAs, especially those of the respiratory and digestive system^25^. Previous studies have discussed digestive system cancers triggered by the ingestion of external agents^26,27^, and the current finding that the most significant cancer risks were observed in the oral, esophagus, and stomach corroborates those earlier findings.

Generally, EAA exposure most commonly occurs via the respiratory system^28^. Accordingly, the particulate characteristics (type or diameter) of the inhaled EAA are an important factor in determining the absorption route and subsequent digestive health effects^29^. Soluble or small particles (1–5 μm) could enter to digestive system by swallowing after secretion via the mucociliary escalator^30^. The ultrafine EAA particles (<1 μm) or insoluble agents can penetrate to the blood or be eliminated by alveolar macrophages and consequently reach the digestive system. Furthermore, man-made EAAs could enter the digestive system via ingestion, including direct hand-to-mouth exposure, in a workplace setting^31^. In summary, all types of EAA can enter the digestive system.

The observation of the highest risks of digestive system cancer in the oral cavity and pharynx may explain the significant relationship between EAA ingestion and the risk of digestive system cancer observed in the current analysis, as both regions are shared by the respiratory and digestive systems. In previous observational cohort studies, male woodworkers exposed to external wood-containing particles had a significantly increased risk of oral and pharyngeal cancer among (SIR = 2.19, 95% CI: 1.17–3.74), and the risk of buccal cavity cancer was even higher than that of pharyngeal cancer^32^. At a cellular level, EAA exposure appears to elevate the risk of chromosomal instability in buccal cells by increasing the frequency of micronuclei, which may induce abnormal cell proliferation or apoptosis^33^. The research has also demonstrated the highest risks of cancer with EAA exposure in the lip, oral cavity, and pharynx. Our current results indicate that the organs at highest risk following exposure to EAA are exposed both via inhalation and ingestion. Therefore, EAA might induce cancer in both the digestive and respiratory pathways.

The esophagus usually functions as a conduit of masticated materials from the oral cavity to the stomach. A previous Japanese cohort study observed a significant increase in the risk of esophageal cancer among patients hospitalized for pneumoconiosis^34^. Similarly, our study demonstrated a significantly increased risk of esophageal cancer among workers with respiratory diseases related to EAA exposure, and the significance of this relationship was not attenuated even after controlling for the effects of smoking and alcohol consumption. These findings suggest a strong link between EAA exposure by ingestion the risk of cancer in initially exposed digestive organ.

Our current investigation also observed an increased risk of stomach cancer in response to EAA exposure. Furthermore, this relationship remained significant in a logistic analysis controlled for smoking and alcohol consumption. These findings broadly support the biomechanics of related studies that have linked gastric cancer with severe respiratory diseases consequent to EAA. We note that most ingested EAAs do not enter the stomach because of the discharge functions of the esophageal sphincter^35^. However, earlier researchers hypothesized that the increased gastric cancer risk due to EAA exposure mostly occurred after lung clearance with or without ingestion process^36,37^, following previous observations of a high risk of gastric cancer among workers who were exposed to external dust. Current study could demonstrate the lowest significant risk for cancer in stomach both SIR and OR. This lowest statistically significant relationship might also attribute to the complexities of gastric cancer and other risk factors, such as *Helicobacter pylori* infection.

Long ago, respiratory system diseases were considered to be the greatest consequences of tobacco smoking exposure, due to beliefs regarding the effects of EAA exposure on the respiratory system. Now, however, the medical field understands that tobacco smoking is also a strong risk factors for digestive system cancer, particularly of upper tract organs^38,39^. Therefore, the effects of both EAA inhalation and ingestion should be determined to fully understand the implications of cancer research. The present study has raised important questions regarding the nature of EAA exposure and its effects on human health. Specifically, EAA ingestion may lead to poorer digestive system health.

The strengths of this study include the large sample size and follow-up design, which allowed us to demonstrate the significant risk of upper digestive tract cancer associated with EAA exposure. However, the study was limited by the availability of only indirect information about EAA exposure. As noted, the NHIS database is based on information from hospital facility visits and is recorded in ICD-10 code format. Accordingly, we could not determine the exact EAA exposure levels. Still, the current investigation focused on the most severe cases of respiratory disease, including all types of pneumoconiosis that could be confirmed as consequences of EAA exposure. Current study only could demonstrate the association between GI tract cancer risk and occupational dust exposed group who were based on extremely severe dust exposure population. Thus, these findings may be somewhat limited to be generalized to the average working population. Furthermore, our study relied on hospital visit information to assess cancer diagnoses and did not obtain pathological confirmation. However, inpatient records involving a major diagnosis of cancer were considered reliable in a previous study that used a similar NHIS data structure^40^. We used different minimum category of cancers. ‘Oral (C00-14)’ cancers and ‘Hepatobiliary tract (C22-26)’ cancers could not possible subgrouping according to ICD-10 three-digit due to very low incidences of each cancers. We hope modified group according to digestive organs might be helpful to understand EEA effect of human organ system. We conducted study with 10-year follow-up duration. Longer observational periods are needed to consider cancer etiology. Finally, the study participants represent a source of uncertainty. As we included only the Korean working population, our findings cannot be extrapolated to a general Korean or global population. Further research should be undertaken to investigate the relationship between EAA exposure and human digestive cancer with general population, specific information of EAA exposure level and cancer, and longer observational periods.

We used ICD-10 codes to assess the EAA exposure, but the J69 (pneumonitis due to solids and liquids) include aspiration pneumonia. Although the workplace exposure such as chemical pneumonitis due to accident related to J69, but the great number of J69 patient suggest that the main cause closely related to aspiration pneumonia. Hence we undertook sensitivity analysis according to excluding or including J69 in EAA exposure group in 1,000,000 random sample date from Korea NHIS. The risk estimates were attenuated when we added J69 as EAA exposure group compare excluding J69 data set (Supplement Table 2). Hence our current result may have underestimating problems.

In conclusion, our large nationwide cohort study revealed that EAA exposure is a risk factor for digestive cancers, particularly oral, esophageal, and stomach cancers. By contrast, no significant EAA exposure-related risk of lower digestive tract cancer and hepatobiliary tract cancer was observed in any analysis. We conclude that cancer researchers should aim to address the risk of digestive cancer following EAA exposure.

## Methods

### Data collection

The NHIS provides mandatory public health insurance for all Korean citizens to cover medical care services consistent with the policies of national health insurance, medical aid, and long-term care insurance^41^. Accordingly, this system covers the entire population residing within the territory of Korea^42^, and all citizens are required by law to participate^43^. The NHIS covered 50,908,646 citizens in 2011, 51,169,141 in 2012, 51,448,491 in 2013, 51,757,146 in 2014, and 52,034,424 in 2015; these values account for approximately 98% of residents in the territory of Korea^44^. For this study, we used data from the NHIS database during the period of 2006–2015.

All types of visits to hospital facilities listed in the Korea NHIS database were categorized using the standardized protocol of the Korea Classification of Diseases and Causes of Death, 4^th^ edition, which corresponds to the International Classification of Diseases, 10^th^ revision (ICD-10). All NHIS claims for inpatient and outpatient visits, procedures, and prescriptions were coded using the ICD-10 format, and the Korean Drug and Anatomical Therapeutic Chemical Codes^45^. The NHIS routinely audits these claims, and the data are considered reliable and have been used in numerous peer-reviewed publications^44^.

The NHIS database additionally includes medical service data qualifications and claims. The qualification data included age, sex, region, income, insurance type, identification number, and family information. The medical service data included records of all covered inpatient and outpatient visits, procedures, and prescriptions. The study also included data from annual medical check-up data for all Korean people, which are provided free of charge by the NHIS and performed annually or biennially to assess chronic disorders, mental health, and lifestyle factors.

### Study participants and definition of the external airborne agent exposure group

EAA exposure first targets the human respiratory system. We therefore defined the EAA exposure group as patients who had ever visited a hospital facility as an inpatient and whose records included ICD-10 codes J60–70, which are categorized as ‘Lung diseases due to external agents’ by the World Health Organization^46^. This category comprises the following 11 sub-codes: J60, Coalworker pneumoconiosis; J61, Pneumoconiosis due to asbestos and other mineral fibers; J62, Pneumoconiosis due to dust containing silica; J63, Pneumoconiosis due to other inorganic dusts; J64, Unspecified pneumoconiosis; J65, Pneumoconiosis associated with tuberculosis; J66, Airway disease due to specific organic dust; J67, Hypersensitivity pneumonitis due to organic dust; J68, Respiratory conditions due to inhalation of chemicals, gases, fumes, and vapors; J69, Pneumonitis due to solids and liquids; and J70, Respiratory conditions due to other external agents. Inpatients with a history including these sub-codes generally experienced an EAA exposure event sufficiently significant to cause a severe respiratory disorder.

In nearly all countries and industries, the main sources of EAAs known to aggravate respiratory system diseases are closely associated with workplace activities, as demonstrated by ICD-10 codes J60–70^47^. These EAAs contain minerals, metals, organic compounds, wood, and cotton and are generated via cutting, milling, grinding, sanding, and cleaning processes. Particularly, pneumoconiosis, respiratory diseases such as hypersensitivity pneumonitis, and airway diseases attributed to any external agents (e.g., mineral fibers, silica, chemicals, or organic/inorganic dust) have been strongly linked with EAA exposure under occupational conditions^48,49^.

99% of EAA exposure was male in current data, therefore, we selected male participants who were employed, aged between 15 and 70 years and were deemed NHIS-eligible employee subscribers from 2006 to 2015 from a total of 122,992,465 person-years. Subsequently, we excluded participants for whom claims for any type of digestive cancer or respiratory disease were recorded from January 1, 2002, to December 31, 2005 (i.e., washout period). Finally, male workers with a total of 79,959,286 person-years were selected for the current study

### Digestive system cancers

Digestive system cancer was defined as the presence of identical ICD-10 C00–26 codes recorded during inpatient visits. Digestive system cancers were classified as oral cancer (C00–14) or gastrointestinal and hepatobiliary tract cancer (C15–26). The latter category was subdivided into gastrointestinal tract cancer (C15–21), which included individual organ cancers (esophageal cancer, C15; stomach cancer, C16; small intestinal cancer, C17; colon cancer, C18; rectosigmoid junction cancer, C19; rectal cancer, C20; and anal and anal canal cancer, C21) or hepatobiliary tract cancer (C22–26).

### Smoking and alcohol consumption status

We evaluated the participants’ smoking and alcohol consumption statuses as potential risk factors for digestive system cancer using national health screening examination data collected during follow-up period. A total 300,290 male workers participated in medical examinations among final study participants. The smoking status was stratified into two classes: none and previous smokers, and current smokers. The alcohol consumption status was similarly stratified into two classes: none and mild drinking, and heavy drinking.

### Statistical methods

Inpatient admission records were used to define incident cases of cancer. We note that because cancer is a chronic nature illness, a single patient will likely be admitted for multiple hospital visits, and the reason for each subsequent hospitalization may differ from the initial admission. In such cases, the first admission was considered the first event and counted as only one case of the indicated type of malignancy. Hence, incidence is defined as the first inpatient admission event in the current study. The person-years and follow-up period were also calculated using the same logic.

We calculated the SIR of each digestive system cancer in the EAA exposure group with reference to a general male working population without admission history of EAAs. Specifically, we first calculated the total observed number of incident cases and age-stratified person-years in the EAA exposure group. Second, we calculated the age-specific incident rates from the reference population and multiplied these values by each age-stratum of person-years in the EAA exposure group to generate the expected counts. The SIR was thus defined as the ratio of the sums of the observed and expected counts. The associated 95% CI was calculated using a Poisson distribution.

To estimate the effects of smoking and alcohol consumption on the risks of digestive system cancers, we conducted logistic regression with OR and 95% CI for the EAA exposure group adjusting for the age, smoking, and drinking status. The smoking and alcohol consumption statuses were available only for those who participated in annual medical heath screening examinations. Smoking and alcohol consumption data used result from latest medical heath screening examinations of each study participants. All analyses were conducted using SAS, version 9.4 (SAS Institute, Cary, NC, USA).

### Ethical consideration

Data of this study was anonymized prior to release to authors from National Health Insurance Service. The Institute Review Board (IRB) of the Yonsei University Health System approved this current study design (IRB number: Y-2017-0100).

## Supplementary information

Supplementary tables.
