# Supplementary material for: External Airborne-agent Exposure Increase Risk of Digestive Tract Cancer
Source: Sci Rep. 2020 May 25;10:8617. doi: 10.1038/s41598-020-65312-6 (PMC7248078; doi:10.1038/s41598-020-65312-6)
Supplement: Supplementary file 1 — Supplementary tables. [file 41598_2020_65312_MOESM1_ESM.docx]

Supplementary information files

**Title: External Airborne-agent Exposure Increase Risk of Digestive Tract Cancer**

**Authors**

Wanhyung Lee^1^, Jihyun Kim^2,3^, Sung-Shil Lim^2,3^, Yangwook Kim^2,3^, Yeon-Soon Ahn^4^, and Jin-Ha Yoon^2,3,5*^

^1^Department of Occupational and Environmental Medicine, Gil Medical Center Gachon University College of Medicine, Incheon, Republic of Korea

^2^The Institute for Occupational Health, Yonsei University College of Medicine, Seoul, Republic of Korea

^3^Graduate School of Public Health, Yonsei University College of Medicine, Seoul, Republic of Korea

^4^Department of Preventive Medicine, Wonju College of Medicine, Yonsei University, Wonju, Korea

^5^Department of Preventive Medicine, Yonsei University College of Medicine, Seoul, Republic of Korea

Supplementary table 1. Number of study participants according to External Airborne agent exposure from 2006 to 2015

| Year | External Airborne agent exposure | | Total |
| --- | --- | --- | --- |
|  | No | Yes |  |
| 2006 | 6,617,562 | 10,794 | 6,628,356 |
| 2007 | 7,015,566 | 10,868 | 7,026,434 |
| 2008 | 7,361,946 | 10,744 | 7,372,690 |
| 2009 | 7,561,585 | 10,335 | 7,571,920 |
| 2010 | 7,803,954 | 9,979 | 7,813,933 |
| 2011 | 8,130,885 | 9,758 | 8,140,643 |
| 2012 | 8,433,199 | 9,495 | 8,442,694 |
| 2013 | 8,691,484 | 9,206 | 8,700,690 |
| 2014 | 9,009,643 | 8,922 | 9,018,565 |
| 2015 | 9,234,796 | 8,565 | 9,243,361 |
| Total | 79,860,620 | 98,666 | 79,959,286 |

| Age-standardized incidence ratio (SIR) and 95% confidence intervals (CI) of cancer of digestive systems among external airborne agent (EAA) exposure group with the national health insurance service–national sample cohort. The sensitivity analysis excluding J69 from EAA exposure group | | | | |
| --- | --- | --- | --- | --- |
| Cancer type (ICD-10) | J60-J70 | | J60-J70 (excluding J69) | |
|  | SIR | 95% CI | SIR | 95% CI |
| All (C00-26) | 1.03 | (0.90-1.15) | 1.26 | (0.99-1.53) |
| Oral (C00-14) | **1.48** | **(1.08-1.87)** | **1.75** | **(1.01-2.87)** |
| Gastrointestinal and hepatobiliary tract (C15-26) | 0.95 | (0.83-1.08) | 1.24 | (0.99-1.53) |
| Gastrointestinal tract (C15-21) | 1.01 | (0.86-1.17) | **1.36** | **(1.06-1.72)** |
| Esophagus (C15) | **2.24** | **(1.10-3.37)** | **3.68** | **(1.61-7.29)** |
| Stomach (C16) | 1.16 | (0.92-1.41) | 1.31 | (0.91-1.84) |
| Small intestine (C17) | 1.71 | (0.01-4.10) | 2.56 | (0.19-12.65) |
| Colon (C18) | 0.78 | (0.53-1.14) | 1.20 | (0.70-1.94) |
| Rectosigmoid junction (C19) | 0.86 | (0.02-1.70) | 2.01 | (0.51-5.48) |
| Rectum (C20) | 0.80 | (0.21-1.39) | 1.07 | (0.50-2.04) |
| Anus and anal canal (C21) | 0.74 | (0.40-1.08) | 0 | NA |
| Hepatobiliary tract (C22-26) | 0.82 | (0.59-1.04) | 0.96 | (0.60-1.48)- |

EAA exposure group : 2,795 (J69: 1,706), Non-EAA exposure group : 770,217 , Cases of GI tract cancer: 28,287
